# GPCR structural characterization by NMR spectroscopy in solution: GPCR structural characterization by NMR

**DOI:** 10.3724/abbs.2022106

**Published:** 2022-08-22

**Authors:** Lingyun Yang, Dongsheng Liu, Kurt Wüthrich

**Affiliations:** 1 iHuman Institute ShanghaiTech University Shanghai 201210 China; 2 Department of Integrative Structural and Computational Biology Scripps Research La Jolla CA 92037 USA; 3 Institute of Molecular Biology and Biophysics ETH Zürich Otto-Stern-Weg 5 8093 Zürich Switzerland

**Keywords:** G protein-coupled receptor dynamics, stable-isotope labeling, fluorine-19 NMR, GPCR biology, drug development

## Abstract

In the human proteome, 826 G-protein-coupled receptors (GPCRs) interact with extracellular stimuli to initiate cascades of intracellular signaling. Determining conformational dynamics and intermolecular interactions are key to understand GPCR function as a basis for drug design. X-ray crystallography and cryo-electron microscopy (cryo-EM) contribute molecular architectures of GPCRs and GPCR-signaling complexes. NMR spectroscopy is complementary by providing information on the dynamics of GPCR structures at physiological temperature. In this review, several NMR approaches in use to probe GPCR dynamics and intermolecular interactions are discussed. The topics include uniform stable-isotope labeling, amino acid residue-selective stable-isotope labeling, site-specific labeling by genetic engineering, the introduction of ^19^F-NMR probes, and the use of paramagnetic nitroxide spin labels. The unique information provided by NMR spectroscopy contributes to our understanding of GPCR biology and thus adds to the foundations for rational drug design.

## Introduction

G protein-coupled receptors (GPCRs) are seven-transmembrane helix (TM) proteins which regulate cellular responses to extracellular stimuli, such as hormones and neurotransmitters [ 1– 3]. With 826 members in the human proteome, GPCRs represent one of the largest membrane protein families. GPCRs have been classified into five main classes, i.e., the rhodopsin (class A), secretin (class B), glutamate (class C), frizzled (class F) and adhesion GPCR families [4]. They are found in almost all human tissues and organs, and they are important drug targets because of their key physiological roles. It was estimated that over 700 approved prescription drugs target GPCRs, implying that approximately 35% of all approved drugs target GPCRs [5]. Structural information on GPCRs therefore has an important role as a foundation for “structure-based rational drug discovery”.

**Figure 1:**
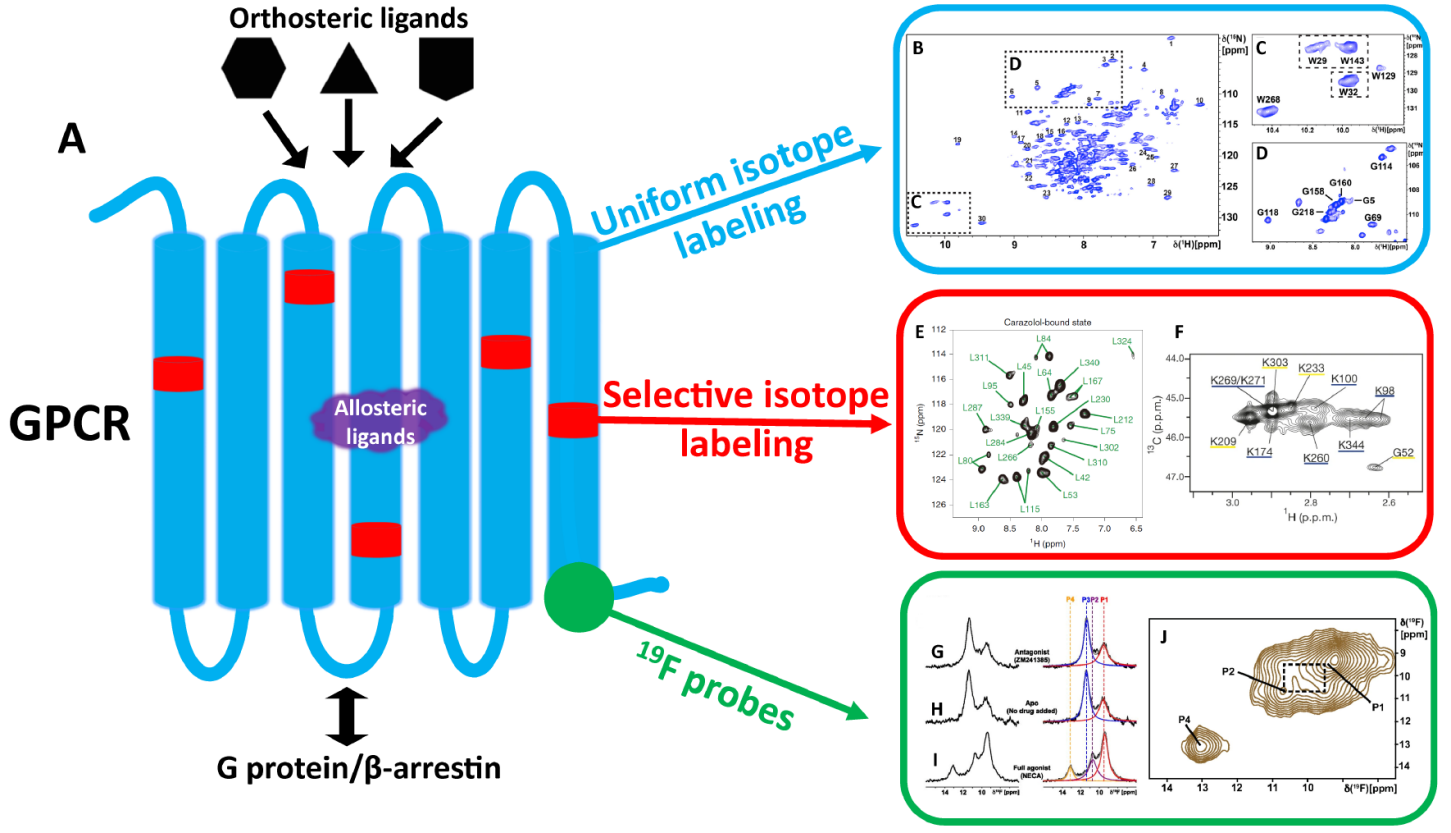
Figure 1 GPCR molecular structure and strategies for NMR characterization (A) Schematic view of a GPCR molecular structure and its interactions with orthosteric and allosteric small molecule ligands (potentially druggable binding sites) and intracellular partner proteins. The black triangle, pentagon and hexagon represent different orthosteric ligands, which all target the orthosteric binding site on the extracellular surface. The purple polygon represents allosteric ligands, which may target a variety of binding sites in GPCR structures. Three NMR spectroscopy approaches to study conformational dynamics and intermolecular interactions of GPCRs are indicated on the right, i.e., uniform stable-isotope labeling (blue, B–D), residue-selective stable-isotope labeling (red, E–F) and 19F-NMR probes (green, G–J). In the GPCR structure, thin blue lines represent extracellular and intracellular loops, thick blue lines are the TMs, red spots and a green circle indicate sites for selective labelling with 13C or 15N, and with a 19F-label, respectively. (B–D) 2D [15N, 1H]-transverse relaxation optimized spectroscopy (TROSY) of A2AAR in complex with the antagonist ZM241385. (E) 2D [15N, 1H]-TROSY spectrum of [2,3,3-2H, 15N]-leu-labeled β2AR. (F) 2D [13C, 1H]-heteronuclear multiple quantum correlation (HMQC) spectrum of ε-N[13CH3]2-lysines in the μ-opioid receptor (μOR). (G–I) 1D 19F-NMR spectra of A2AAR[A289CTET] bound to an inverse agonist, in the apo-form, and bound to an agonist. Spectral components contained in the observed signal envelope are shown on the right of this panel. (J) 2D [19F, 19F]-EXSY spectrum of the A2AAR–agonist complex in I, recorded at 280 K with a mixing time of 100 ms. B to D are adapted from Figure1 in reference [20]. E is adapted from Figure1 in reference [23]. F is adapted from Figure1 in reference [28]. G to J are adapted from Figure1 in reference [29].

GPCRs interact with small molecule ligands as well as intracellular partner proteins [6]. Ligands binding to the orthosteric site on the extracellular GPCR surface ( Figure 1A) have been extensively studied and represent nearly all approved drug molecules. Orthosteric ligand binding has been shown to cause major conformational changes of GPCRs. The molecular architecture can be seen by crystallography or electron microscopy, while local conformational polymorphisms have in many cases been observed by NMR spectroscopy [7]. Generally, agonist-bound GPCRs mediate cellular responses by interacting with intracellular proteins such as G proteins and β-arrestins. Dual pathways of G protein and β-arrestin coupling have been described in a wide variety of *in vitro* and *in vivo* systems [ 8, 9]. Biased ligands preferentially activate one of the signaling pathways, which offers potential for reducing drug side effects and safety issues [10].

Allosteric ligands bind spatially distinct from the orthosteric pocket and can serve as additional tools to modulate function-related conformational states of the GPCRs. Positive allosteric modulators (PAMs) increase the response of the receptor to a given orthosteric ligand, while negative allosteric modulators (NAMs) attenuate receptor activity [11]. Allosteric modulators approved as drugs include cinacalcet (PAM of a calcium-sensing receptor) and maraviroc (NAM of the chemokine receptor CCR5) [12]. Overall, allosteric ligands thus enrich the ways to manipulate the functions of GPCRs for potential therapeutic benefits [13].

## NMR Methods for the Study of GPCRs

Since the molecular architecture of the human β _2_-adrenergic receptor (β _2_AR) was reported in 2007 [14], more than 400 structures of human GPCR complexes have been determined by X-ray crystallography [15]. During the past few years, cryo-electron microscopy contributed structure determinations of GPCR signaling complexes including orthosteric ligands and G proteins or β-arrestins. Recently, AI methods have generated GPCR structures with atomic accuracy even in cases in which no similar structure is known [ 16, 17]. NMR spectroscopy technologies complement the information on the molecular architectures obtained by these methods, especially with studies of the structural dynamics and of intermolecular interactions at physiological temperatures. Gautier *et al*. [18] also reported a *de novo* NMR structure determination of the phototaxis receptor sensory rhodopsin II (pSRII). With the use of transverse relaxation-optimized spectroscopy (TROSY) [19], it is quite generally possible to obtain *de novo* structure determination of GPCRs. However, NMR spectroscopy approaches to study structural dynamics of GPCRs and intermolecular interactions with GPCRs complement molecular architectures determined with different methods, and this type of NMR applications is pursued by many groups around the world.

### NMR studies using uniform stable-isotope labeling

Uniform stable-isotope labeling is a widely-used method for structural studies of proteins. ^15^N and ^13^C are enriched in the protein far over the natural abundance to increase the sensitivity ( Table 1) and overcome the ^1^H signal overlapping problem of NMR spectroscopy with biological macromolecules. Eddy *et al*. [20] expressed A _2A_ adenosine receptor (A _2A_AR) in *Pichia pastoris* to obtain uniform double labeling with ^2^H and ^15^N ( Figure 1B–D). All six tryptophan side-chain and eight glycine backbone ^15^N- ^1^H NMR signals were assigned by engineered amino acid replacements. The uniform labeling provided a comprehensive view of plasticity and structural dynamics of the entire receptor. Egloff *et al*. [21] and Nasr *et al*. [22] used directed evolution technologies to obtain rat neurotensin receptor 1 (NTR1) in *E*. *coli*, which was then reconstituted in circularized nanodiscs for collecting [ ^15^N, ^1^H]-TROSY spectra. Uniform stable-isotope labeling can afford, in principle, a comprehensive view of GPCR structural dynamics, but obtaining NMR assignments of GPCRs tends to be a major challenge [20]. Limitations arise because of the high cost of ^13^C, ^15^N, ^2^H triple-labeling and low expression levels. Furthermore, the molecular weight of detergent-solubilized complex of GPCRs is close to 100 kDa, which leads to increased line widths and signal overlaps in the NMR spectra.

**Table:** **Table 1** Nuclear properties of selected isotopes

NMR isotopes	Spin	Natural abundance (%)	Gyromagnetic ratio (10 ^7^ rad s ^−1^ T ^−1^)	Sensitivity rel. ^1^H ^a^
^1^H	1/2	99.99	26.7522	1.00
^13^C	1/2	1.07	6.7282	1.59×10 ^−2^
^15^N	1/2	0.36	−2.7126	1.04×10 ^−3^
^19^F	1/2	100	25.1623	0.83

From Bruker topspin user guide, BRUKER Almanac. ^a^ For work at natural abundance, the effective sensitivity for NMR detection relative to ^1^H is obtained by multiplying the “sensitivity” with the “natural abundance” (in %).

### NMR studies using amino acid residue-selective stable-isotope labeling

Residue-selective stable-isotope labeling reduces peak overlap in the NMR spectra, when compared with uniform stable-isotope labeling. Imai *et al*. [23] used [2,3,3- ^2^H, ^15^N]-leucine to selectively label β _2_AR in insect cells ( Figure 1E). Assignments were established by engineered amino acid replacements, similar to the aforementioned illustration of uniform stable-isotope labeling [20]. Upon binding of the agonist formoterol, chemical shift differences larger than 0.4 ppm were observed for L212, L284 and L287, indicating that large local conformational changes of the PIF motif region are associated with activation. In addition to ^15^N, ^13^C enrichment has also been used for residue-selective stable-isotope labeling, such as ^13^C-dimethylated lysine [ 24, 25], and ^13^CH _3_-ε-methionine [ 26, 27]. Sounier *et al*. [28] expressed the μ-opioid receptor (μOR) in *sf*9 and applied posttranslational reductive ^13^C-methylation to methylate the ε-NH _2_ groups of lysine side chains (ε-N[ ^13^CH _3_] _2_-lysines). Figure 1F shows the [ ^13^C, ^1^H]-heteronuclear multiple quantum correlation (HMQC) spectrum of the ^13^C labeled μOR. Comparisons of the spectra of μOR with different ligands bound indicated that conformational changes in TM5 and TM6 are almost completely dependent on the presence of both the agonist and a G protein mimetic nanobody. Residue-selective stable-isotope labeling can quite generally yield multiple-site structural dynamics information from NMR spectra with reduced peak overlap, as applied in the illustrations of Figure 1E,F, with GPCRs from eukaryotic expression systems.

### NMR studies using chemically conjugated ^19^F-NMR probes

Although the uniform or residue-selective labeling methods have been greatly optimized, it is challenging for GPCRs to be highly expressed in labeling media for insect cells or mammalian systems. ^19^F is an attractive nucleus with high sensitivity and 100% natural abundance ( Table 1). Furthermore, ^19^F is rarely found in biological macromolecules, which enables the NMR detection of extrinsic ^19^F labels with minimal background signals. ^19^F-probes can be introduced by post-translational chemical modification [30], and they have been widely used for studies of structure and dynamics of proteins during the past several decades [ 31– 34]. As with all extrinsic probes, one has to deal with the concern that fluorine labeling could affect the conformation of the protein. However, indications so far are that GPCR conformations are little affected by carefully designed labeling sites. This is especially true for the post-translational conjugation of the fluorine labels, since only solvent-exposed residues are readily labeled. Proper functional assays and combination of ^19^F-NMR with other biophysical and biochemical methods help to verify that structure and function of the receptor are preserved after labeling with fluorine probes.

**Figure 2:**
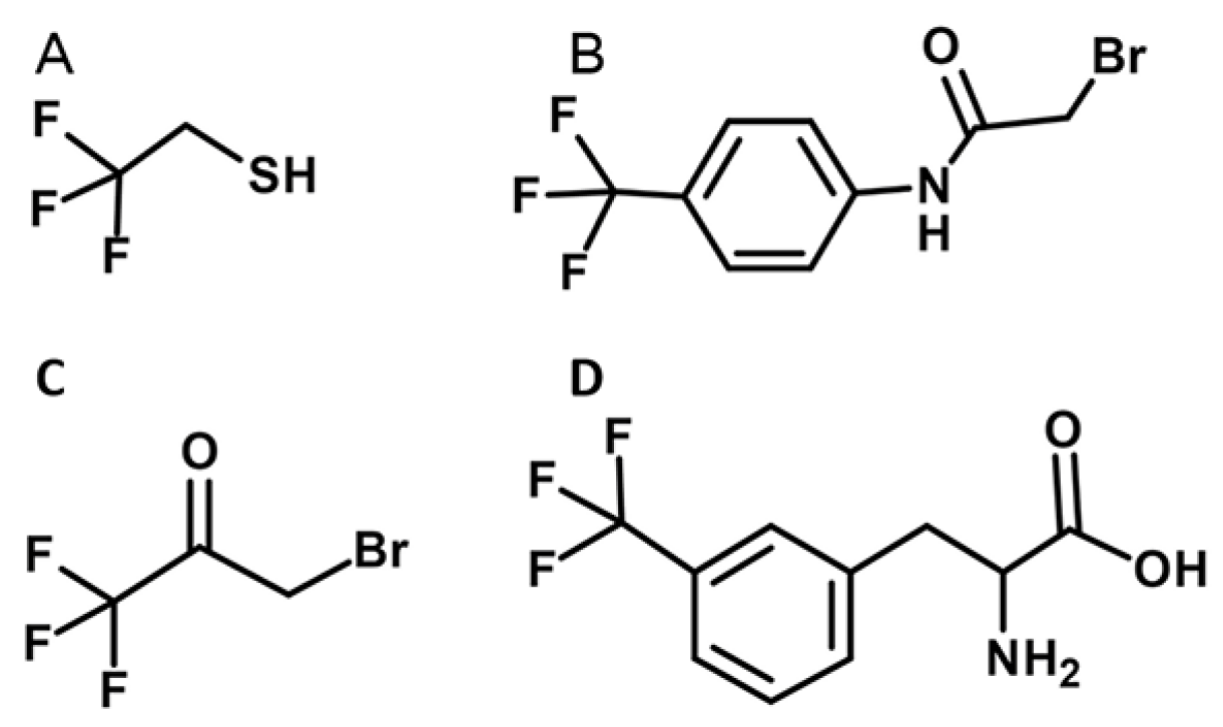
Figure 2 Chemical structures of ^19^F-NMR probes used for studies of GPCRs (A) 2,2,2-trifluoroethanethiol (TET). (B) 2-bromo-N-(4-(trifluoromethyl) phenyl) acetamide (BTFMA). (C) 3-bromo-1,1,1-trifluoroacetone (BTFA). (D) 3-trifluoromethyl-L-phenylalanine (mtfF).

Figure 2 presents the chemical structures of ^19^F-NMR probes that have been used for studies of GPCRs. The sizes of 3-bromo-1,1,1-trifluoroacetone (BTFA) and 2,2,2-trifluoroethanethiol (TET) are small, causing minimal structural perturbations [30]. BTFA, which can form a stable thioester bond with the protein by a one-step process, has been used in β _2_AR studies [35]. Similarly, TET, which can link with cysteines by a disulfide bond, has been used in β _2_AR and A _2A_AR [ 36, 29]. 2-bromo-N-(4-(trifluoromethyl) phenyl) acetamide (BTFMA) exhibits an outstandingly large range of chemical shift differences with variable solvent polarity [37], and it has been used for interaction studies of A _2A_AR with G-proteins [38].

In a historical first, Klein-Seetharaman *et al*. [39] applied TET to successfully label rhodopsin in detergent micelles. Six single cysteine mutants of rhodopsin were labeled and studied with ^19^F-NMR in dark environment and after illumination. Clear chemical shift changes demonstrated the applicability of solution ^19^F-NMR spectroscopy for studies of different rhodopsin activation states in the dark and on light activation. TET labels were also introduced into β _2_AR, and ^19^F-NMR revealed equilibria between simultaneously populated, locally different conformational states near the cytoplasmic receptors surface [36]. While agonist binding primarily shifts the equilibrium towards the G protein-specific active state, the β-arrestin-biased ligands predominantly impact the other conformational state. 2D [ ^19^F, ^19^F] exchange spectroscopy (EXSY) and 1D ^19^F saturation transfer NMR experiments were then used to further investigate the exchange rates between the two conformational states [40]. Sušac *et al*. [29] successfully introduced TET probes to the intracellular surface of A _2A_AR, showing largely different 1D ^19^F-NMR spectra for A _2A_AR[A289C ^TET^] bound to an inverse agonist, in the apo-form, and bound to an agonist ( Figure 1G–I). Two signals with chemical shifts of 11.4 ppm and 9.5 ppm were observed in the apo-form and the antagonist bound state of A _2A_AR, representing two conformational states. In the active-like state of the agonist complex, two new peaks at 10.8 ppm and 13.1 ppm appeared, indicating a transition to a different conformational state. EXSY cross peaks ( Figure 1J) then further demonstrated conformational exchange between the different activation levels. These results suggest that A _2A_AR activation includes both induced fit and conformational selection mechanisms. In addition, the comparison of A _2A_AR and a constitutively active mutant established correlations between NMR parameters and GPCR basal activity.

These works nicely illustrate advantages of ^19^F-NMR probes for studies of conformational dynamics of GPCRs. However, care needs to be exercised to account for possible non-specific labeling with ^19^F-NMR probes [ 35, 41]. In-membrane chemical modification (IMCM) was developed to obtain selective chromophore labeling of intracellular surface cysteines in GPCRs with minimal mutagenesis [42], which greatly reduced the non-specific labeling problem encountered in detergent micelles.

### NMR studies using genetically incorporated ^19^F-NMR probes

Most of the human GPCRs harbor more than 10 cysteine residues, and mutation of all surface-exposed cysteine residues for the prevention of non-specific labeling may cause significant structural perturbation. Moreover, residues buried in the protein core cannot usually be labeled through the chemical conjugation approach described in the preceding section. Genetic incorporation of fluorine-containing unnatural amino acids can overcome these limitations of the cysteine conjugation methods, which ensures that the modified protein can be expressed with sufficiently high yields.

**Figure 3:**
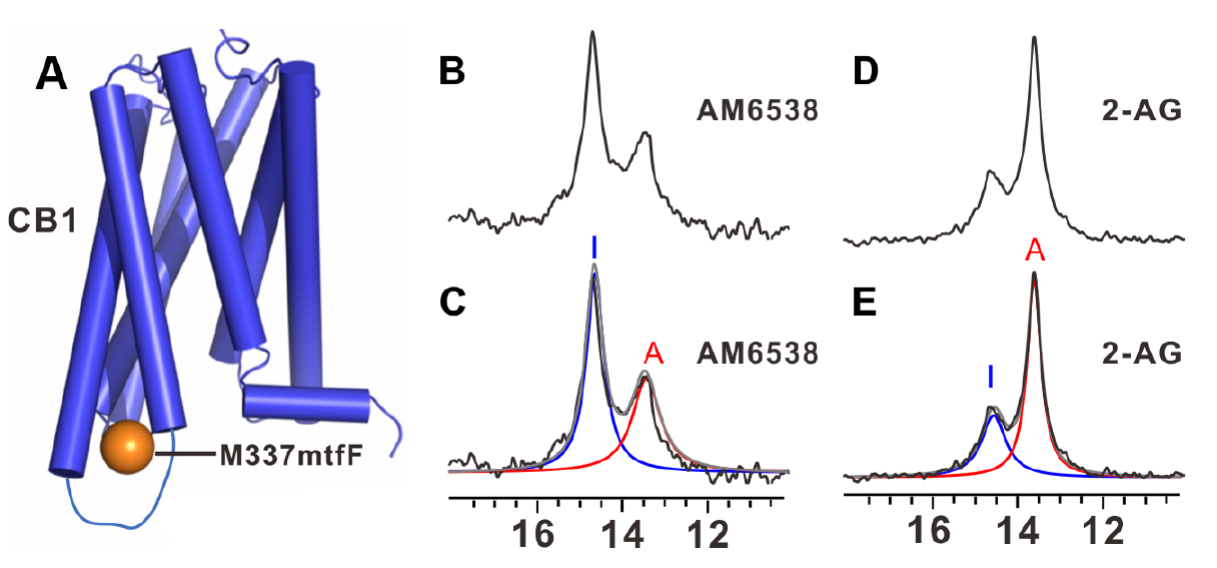
Figure 3 Schematic view of cannabinoid receptor 1 (CB1) with ^19^F-NMR probe and its ^19^F-NMR spectra (A) Schematic view of CB1 with a genetically engineered 19F-NMR probe, 3-(trifluoromethyl) phenylalanine (mtfF) at the sequence position 3376.29. The brown sphere represents the position of the mtfF. (B,D) 1D 19F-NMR spectra of CB1 [M3376.29mtfF] in complexes with the antagonist AM6538 and the agonist 2-AG, respectively. (C,E) Spectra of B,D, respectively, with indication of the component signals (blue and red) identified by Lorentzian deconvolution. The spectra B to E were previously included in Figure2 of reference [47].

^19^F-containing phenylalanine and tyrosine analogs have been incorporated into proteins in prokaryotic expression system [ 43– 46]. In 2021, Wang *et al*. [47] reported successful genetic incorporation of 3′-trifluoromethyl-L-phenylalanine (mtfF, see Figure 2D) into the cannabinoid receptor 1 (CB1), using the baculovirus *sf*9 expression system. Figure 3A is a schematic view of CB1 with this genetically engineered mtfF probe at the sequence position 337. Figure 3B,D shows the 1D ^19^F-NMR spectra of CB1 [M337 ^6.29^mtfF] in complexes with the antagonist AM6538 and the agonist 2-AG, respectively. Two ^19^F-NMR peaks at 14.6 ppm (I) and 13.5 ppm (A) represent inactive and active-like states, respectively. Effects of the allosteric modulator Org27569 on the conformational landscape of CB1 were also characterized. Using this approach for investigating site-specific dynamics of GPCRs adds to the great potential of ^19^F-NMR probes in eukaryotic proteins. With genetically incorporated ^19^F-NMR probes, the peak assignment is straightforward, and the aforementioned non-specific labeling problem with chemical conjugation methods is, in principle, not encountered.

### NMR studies using paramagnetic nitroxide spin labels

Paramagnetic relaxation enhancement (PRE) is often used for studying long-distance intra- or intermolecular interactions [ 48– 50]. The unpaired electron spin (such as a nitroxide spin-label or paramagnetic metal ions) enhances the relaxation rates and may affect the chemical shifts of nearby nuclear spins. The PRE decreases proportional to r ^–6^ (where r is the distance between the paramagnetic center and the nuclear spin), and the effects over distances up to about 20 Å can be observed with nitroxide spin labels [51]. Using the spin-label, S-(2,2,5,5-tetramethyl-2,5-dihydro- ^1^H-pyrrol-3yl) methyl methanesulfonothioate (MTSL) introduced by chemical coupling, Imai *et al*. [23] evaluated the distance between a particular amide proton and this paramagnetic center in β _2_AR. In addition, the solvent accessibilities of residues in GPCRs could be examined by solvent PRE experiments. Takuya *et al*. [52] used Gd-diethylenetriamine pentaacetic acid-bismethylamide (Gd-DTPA-BMA), which is a highly water-soluble paramagnetic probe, to investigate the solvent accessibilities of the methionine residues in [[α,β,β- ^2^H, methyl- ^13^C] Met, u- ^2^H] A _2A_AR. From the NMR studies, the authors concluded that the A _2A_AR conformation was shifted toward states that are preferable for G protein binding. Similar paramagnetic Gd ^3+^-DTPA probe was used by Lindsay *et al*. [53] to detect solvent accessibility. The solvent PRE effects seen for Ile δ1- ^13^C-labeled A _2A_AR indicated that breathing of the structure to expose this region to the Gd ^3+^-DTPA complex. Combined with the aforementioned stable-isotope labeling methods of GPCRs, PRE opens avenues to a wide range of novel strategies.

## Conclusions and Perspectives

With the use of various labeling methods, NMR spectroscopy is an attractive technique in structural biology of GPCRs. NMR spectroscopy is used to explore conformational dynamics and intermolecular interactions to complement the molecular architectures obtained from X-ray crystallography and cryo-electron microscopy [7]. *De novo* structure determination of GPCRs with NMR spectroscopy in solution has also been described [18], but this is currently not a focus and no similar work has recently appeared. Among the various labeling approaches, ^19^F-NMR probes have outstanding potential to investigate conformational equilibria and associated rate processes with high sensitivity. Genetic site-specific ^19^F-labeling technique in eukaryotic expression systems promises to become a powerful tool for studies of otherwise not detectable conformational states of GPCRs. With future improvements of biochemical and biosynthetic methods, for uniform or selective stable-isotope labeling as well as for the introduction of ^19^F-probes and paramagnetic nitroxide spin labels, NMR spectroscopy in solution will further strengthen its role in GPCR structural biology.
